# Birthweight predicts adult cardiovascular disorders: Population based cross sectional survey

**DOI:** 10.1002/clc.23419

**Published:** 2020-07-29

**Authors:** Issa Salmi, Suad Hannawi

**Affiliations:** ^1^ The Medicine Department The Royal Hospital Muscat Oman; ^2^ The Medicine Department MOHAP Dubai UAE; ^3^ Oman Medical Specialty Board Muscat Oman

**Keywords:** cardiovascular, epidemiology, general clinical cardiology/adult, heart failure, ischemic heart disease, myocardial infarction, pathophysiology of cardiac disease, preventive cardiology, stroke prevention, women

## Abstract

**Background:**

Cardiovascular disease (CVD) is the primary cause of death in the developed‐countries and mostly in the poorer areas of the country, and in lower income‐groups.

**Hypothesis:**

Birthweight predicts adult development of angina, coronary heart disease, stroke, and combination of all CVD.

**Methods:**

The AusDiab is a cross‐sectional study of Australians aged 25 years or over. Data on age, sex, previous‐CVD, smoking‐status, alcohol‐intake, time‐spent on watching television and physical‐activity, total house‐income, dwelling‐type and education‐level were collected by interviewer‐ administered‐questionnaires.

**Results:**

Four thousand five hundred and two had birthweights (mean (SD) of 3.4(0.7) kg). Females in the lowest birthweight‐quintile were at least 1.23, 1.48, 1.65, and 1.23 times more likely to have angina, CAD, stroke, and CVS compared to the referent group ≥3.72 kg with *P* = .123, .09, .099, and 0.176, respectively. Similarly, males in the lowest‐birthweight‐quintile were 1.23, 1.30, 1.39, and 1.26 times more likely to have angina, CAD, stroke, and CVS compared to the referent‐group ≥4.05 kg with *P* = .231, .087, .102, and .123, respectively.

Females with low birth weight (LBW) were at least 1.39, 1.40, 2.30, and 1.47 times more likely to have angina, CAD, stroke and CVS compared to those ≥2.5 kg with *P* = .06, .19, .03, and .13, respectively. Similarly, males with LBW were 1.76, 1.48, 3.34, and 1.70 times more likely to have angina, CAD, stroke, and CVS compared to those ≥2.5 kg with *P* = .14, .13, .03, and .08, respectively.

**Conclusion:**

there was a negative relationship between birth weight and angina, CAD, stroke, and the overall CVS. It would be prudent, to adopt policies of intensified whole of life surveillance of lower‐birthweight people, anticipating this risk.

**Abbreviations**
BMI: body mass index
BW: birth weight
CAD: coronary artery disease
CHD: coronary heart disease
CVD: ardiovascular disease
CVS: cardiovascular system abnormalities
LBW: low birth weight
NCD: noncommunicable diseases

## 1 INTRODUCTION

The prenatal‐environment of babies in the women womb is of paramount importance in adult‐life illnesses hazard and it may impact on the later rise of noncommunicable chronic diseases. The birth weight may be considered as a crude assessment of the prenatal‐environment circumstances in the uterus. Low birth weight (LBW), a consequence of poor prenatal environment contributes to this trend of disease programming in early on adult natural life cycle. Various noncommunicable disease (NCD) in human is controlled by genetic and environmental factors early in our life cycle. The manner in which gene representation may be forever changed by the prenatal environment such as the dietary environment or various toxic effects or deprivations in early life and hence it is not only the presence or absence of genes that control the risk for NCD development. Researchers findings hint that the threat of chronic‐diseases in adult natural life is coded, programmed, and/or imprinted by the prenatal environment in utero.^1^, ^2^, ^3^, ^4^, ^5^, ^6^

Cardiovascular disease (CVD) is the primary cause of death in the developed countries and mostly in the poorer areas of the country, and in lower income groups. Barker et al found that distribution of deaths occurred from coronary heart disease (CHD) across England and Wales during 1968 to 1978 closely resembled the distribution of infant deaths.^7^ Geographical studies yielded the first indication that CVD may originate from prenatal live during intra‐uterine development. Barker DJ clearly found that variations in mortality from the disease across England and Wales were shown to correlate closely with past differences in death rates among newborn babies.^8^

Subsequently, it was found that cardiovascular mortality in later life is related to LBW in men and women born in Hertfordshire, United Kingdom, between 1911 and 1930.^9^ This association between LBW and subsequent risk of CVD was confirmed by other studies from UK, USA, and Europe.^10^, ^11^, ^12^, ^13^, ^14^, ^15^, ^16^, ^17^, ^18^, ^19^, ^20^ These studies, however, were performed in selected populations, based on geographical location, ethnicity, and/or professional status. None have looked at the phenomenon in a general adult population.

Some researchers had criticized the fetal origin hypothesis on grounds that many had obtained significant results after adjusting for current body weight and or that physical activity, smoking status, alcohol intake, family history, and socioeconomic status were not taken into consideration when examining relationships.^21^, ^22^, ^23^, ^24^, ^25^ In addition, some results were not reported separately for females and males.

Taking these factors in consideration, we evaluated the relationship between birthweight and angina, CHD, and stroke in the general population.

## 2 METHODS

### 2.1 Participants

The AusDiab survey is a cross sectional study in which data were collected from a stratified sample of Australians aged 25 years or over, residing in 42 randomly selected urban and nonurban areas (Census Collector Districts) of the six states of Australia and the Northern Territory.^26^ At our instigation, questions about birthweight were added to the second round of the AusDiab study. Participants were asked to state what their birthweight was. Then, participants were asked about the accuracy of the stated birthweight. This was followed by a question about the source of their stated birthweight.

### 2.2 Measurements

Detailed methodology of the AusDiab study had been discussed in a previous manuscript.^26^ In brief, data on age, sex, previous CVD (angina, CHD, and stroke), smoking status, alcohol intake, time spent on watching television and physical activity, total house income, dwelling type, and education level were collected by interviewer‐administered questionnaires.^27^, ^28^, ^29^ All subjects attended a local screening venue and completed a series of questionnaires, physical examinations, and specific laboratory tests which examined diabetic status, cardiovascular risk factors, and kidney function. An interviewer‐administered questionnaire was used to determine smoking status, alcohol consumption, leisure‐time physical activity, and television viewing. Assessment of socioeconomic status was based on education, dwelling type, and income.

Participants self‐reported their frequency and duration of physical activity during the previous week. Physical activity was measured by the Active Australia questionnaire, which asks respondents about their participation in predominantly leisure‐time physical activities (including walking for transport).^30^ These questions have been found to provide reliable and valid estimates of adult physical activity.^30^ Total physical activity time was calculated as the sum of the time spent walking (if continuous and for ≥10 minutes) or performing moderate‐intensity physical activity, plus double the time spent in vigorous‐intensity physical activity.^31^ Frequency of physical activity was calculated by summing the number of sessions of vigorous activity, moderate activity, and walking. Physical activity was categorized to reflect the current Australian public health recommendation for physical activity^31^ as active (≥150 minutes/week across at least five sessions) and inactive (<150 minutes/week and/or fewer than five sessions).

Participants also self‐reported the total time they spent watching television (TV) or videos in the previous week. This measure provides a reliable and valid estimate of TV time among adults.^32^ The average hours watching TV per week were used to create three categories of TV viewing (0‐7, 7.01‐14, and >14 hours/week).

During the 2004 to 2005 follow‐up AusDiab survey, questions about birthweight were included. Participants were asked to state their birthweight, the likely accuracy of the stated birthweight and the source of their stated birthweight. Birthweights were recorded as pounds and ounces or in kilograms (kg) and grams. All values were converted to kilograms for analyses. LBW is defined by the World Health organization (WHO) as a birth weight of an infant of 2499 g or less (<2.5 kg).^33^ Participants were also divided into sex‐specific birthweight quintiles (about 900 participants in each group, females: 542 in each group and males: 358 in each group) for further categorical analyses. This end up as lowest birth weight quintile was <2.81 and <3.06 for females and male, respectively. The highest birth weight quintile was >3.72 and ≥4.05 for females and male participants, respectively. Participants with angina and/or CAD and/or stroke were grouped together as suffering from CVD.

All subjects underwent height/weight measurement except those who were (a) chairbound, (b) pregnant, or (c) too unsteady on their feet. Height was measured to the nearest 0.5 cm without shoes using a stadiometer. Each participant stood fully erect on a firm, flat surface with heels, buttocks, and shoulders resting lightly against a backing board so that the Frankfort plane (a line connecting the superior border of the external auditory meatus with the infraorbital rim) was horizontal (ie, parallel to the floor).

Weight was measured on a firm, flat surface without shoes and excess clothing, using digital weighing scales (Wedderburn Personal Digital Scales TI‐HD316), and was recorded to the nearest 0.1 kg. The accuracy of the scales was checked on a daily basis by using a 5 kg weight. The scales were not able to measure participants who weighed ≥150 kg. body mass index (BMI) was calculated as weight (kg)/height (m)^2^. BMI groups were classified according to World Health Organization criteria^34^ as follows: Normal <25.0 kg/m^2^, Overweight 25.0 to 29.9 kg/m^2^,and Obese ≥30.0 kg/m^2^_._

### 2.3 Statistics

Characteristics of the study sample are described by numbers and percentages for categorical variables and mean (SD) for continues variables. Student *t*‐test was used for normally distributed variables. Logistic regression was used to assess the strength of the relationship of angina, coronary artery disease (CAD), stroke and all cardiovascular system abnormalities (CVS) to birthweight. Significance was considered at *P* < .05. We examined the relationships for each gender separately except when examined by BMI categories, data were combined for both sexes as the number were small. We adjusted for age, adjusted for age, and body mass and for various confounding factors that include body mass, physical activity (based on time spent on exercise and watching television), smoking status, alcohol intake, and socioeconomic status. Stata for windows package software, StataCorp, College Station, Texas 77845 was used for statistical analyses.

## 3 RESULTS

Of the 7157 who responded to our questionnaire, 4502 (63%) reported information related to their birthweight.

**FIGURE 1:**
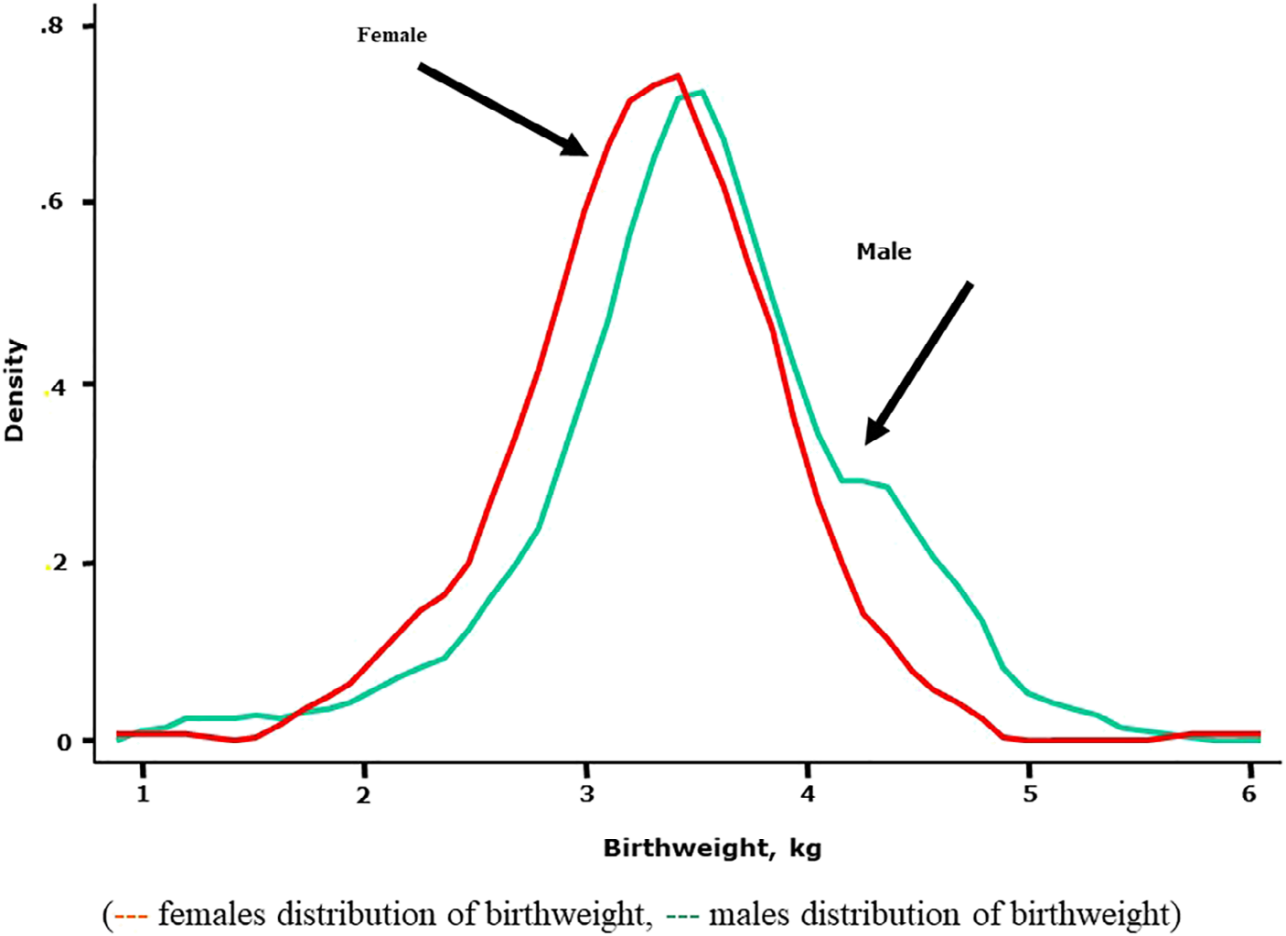
The birthweight distribution of the participants, which ranged from 0.4 to 7 kg with a mean (SD) of 3.37 (0.7) kg

(12) People who reported their birthweight were younger, with a mean (SD) age of 48 (12) vs 51 years for those who did not report their birthweight, *P* < .001. As shown in Figure 1 ^(1)^, The birthweight of the participants ranged from 0.4 to 7 kg with a mean (SD) of 3.37 (0.7) kg. The mean birthweight was lower for females, 3.28 (3.26‐3.31) kg, when compared to males, 3.5 (3.47‐3.53) kg. The prevalence of LBW (<2.5 kg) was 8%, 1 % had a birthweight less than 1.5 kg and 1 % had a birthweight of 5 kg and over.

Of those who provided their birthweight, 141 (3.16%) had angina, 105 (2.35%) had CAD, 62 (1.39%) had stroke, and 143 (3.19%) had one or more of the mentioned cardiovascular disorders.

Table 1 shows proportions of angina, CAD and stroke by birthweight quintiles for females and males. In females with lowest birthweight quintile, the proportions with angina, CAD, stroke and all abnormalities (CVS) were, although was significant only for CAD (*P* = .036), higher than those of higher birthweight. In males with lowest birthweights quintile, the proportions for angina, CAD, stroke, and CVS were higher, but nonsignificant (*P* = .056), than those for higher birthweight.

**TABLE 1:** Proportion of angina, coronary artery disease, stroke, and combined cardiovascular system abnormalities (CVS) by birthweight quintiles

Females	<2.81	2.81‐	3.19‐	3.41‐	>3.72 kg	
Number	546	637	526	466	536	*P*
Angina	3.68 (2.30,5.84)	2.10 (1.20,3.51)	1.72 (0.88,3.27)	2.94 (1.81,4.75)	3.01 (1.85,4.84)	.283
CAD	3.03 (1.80,5.05)	0.79 (0.33,1.89)	0.95 (0.40,2.27)	1.66 (0.87,3.15)	2.07 (1.15,3.69)	.036
Stroke	2.84 (1.91,3.38)	1.59 (0.86,2.93)	1.15 (0.52, 2.03)	1.51 (0.72,3.14)	1.12 (0.51, 2.07)	.184
CVS	3.66 (2.29,5.81)	2.06 (1.20, 3.51)	1.71 (0.89,3.26)	2.94 (1.81,4.74)	3.01 (1.84,4.83)	.285

Males	<3.06	3.06‐	3.37‐	3.64‐	≥4.05 kg	
Number	364	355	408	311	353	*P*
Angina	5.71 (3.72,8.69)	2.59 (1.35, 4.89)	4.70 (3.02,7.25)	2.27 (1.08,4.67)	4.16 (2.52,6.78)	.099
CAD	5.01 (3.26,7.77)	2.29 (1.15,4.52)	3.47 (2.07,5.78)	1.63 (0.68,3.85)	4.56 (2.81,7.31)	.056
Stroke	2.70 (1.77,3.73)	1.15 (0.43,3.01)	0.49 (0.12,1.95)	1.61 (0.67,3.82)	1.66 (0.75,3.64)	.144
CVS	5.95 (3.91,8.95)	2.85 (1.54,5.21)	4.69 (3.01,7.24)	2.26 (1.08,4.66)	4.13 (2.51,6.74)	.100

Table 2 shows the odds ratios (OR, 95%CI) for having angina, CAD, stroke and CVS by birthweight quintiles. Females in the lowest birthweight quintile were at least 1.23, 1.48, 1.65, and 1.23 times more likely to have angina, CAD, stroke, and CVS compared to the referent group ≥3.72 kg with *P* = .123, .09, .099, and .176, respectively. Similarly, males in the lowest birthweight quintile were 1.23, 1.30, 1.39, and 1.26 times more likely to have angina, CAD, stroke, and CVS compared to the referent group ≥4.05 kg with *P* = .231, .087, .102, and .123, respectively. These relationships increased or persisted with adjustments for potential confounding factors. In males with birthweight between 3.06 and 3.26 were at least risks for cardiovascular problems compared to referent group. These relationships persisted when stratified by body mass index as shown in Table 3. After adjustment for age and sex, people in the lowest birthweight quintile, (<2.8 and <3.06 kg for females and male, respectively), had higher risk for developing angina, CAD, stroke and CVS compared to the referent quintile group (≥2.81 and 3.06 kg for females and males, respectively) .

**TABLE 2:** Odds ratios and 95%CI for angina, coronary artery disease (CAD), stroke, and all cardiovascular system abnormalities (CVS) by birthweight quintiles

Females		<2.81	2.81‐	3.19‐	3.41‐	>3.72	
Number		546	637	526	466	536	*P* trend
Angina	a	1.23 (0.62,2.47)	0.68 (0.32,1.42)	0.56 (0.24,1.29)	0.98 (0.48,1.97)	1.0	.123
	b	1.69 (0.81,3.50)	0.84 (0.39,1.81)	0.75 (0.32,1.76)	1.07 (0.51,2.22)	1.0	.09
	c	1.78 (0.95,3.70)	0.91 (0.42,1.96)	0.78 (0.33,1.83)	1.13 (0.54,2.35)	1.0	.07
	d	2.03 (0.97,4.72)	0.85 (0.34,2.12)	0.88 (0.34,2.26)	1.11 (0.47,2.61)	1.0	.06
CAD	a	1.48 (0.67,3.29)	0.38 (0.13,1.09)	0.45 (0.15,1.32)	0.79 (0.32,1.94)	1.0	.090
	b	1.96 (0.86,4.49)	0.46 (0.15,1.37)	0.58 (0.20,1.74)	0.85 (0.34,2.12)	1.0	.06
	c	2.48 (1.05,5.86)	0.47 (0.15,1.56)	0.71 (0.23,2.16)	1.05 (0.41,2.66)	1.0	.001
	d	4.31 (1.39,13.4)	0.44 (0.10,2.36)	0.81 (0.19,3.50)	1.09 (0.31,3.84)	1.0	<.001
Stroke	a	1.65 (0.85,4.57)	1.41 (0.51,3.93)	1.02 (0.32,3.18)	1.35 (0.45,4.04)	1.0	.099
	b	1.78 (0.96,5.01)	1.73 (0.62,4.87)	1.29 (0.41,4.11)	1.71 (0.56,5.22)	1.0	.068
	c	1.99 (0.98,5.35)	1.90 (0.67,5.36)	1.36 (0.43,4.33)	1.83 (0.60,5.58)	1.0	.057
	d	1.95 (0.91,5.42)	1.41 (0.41,4.83)	1.10 (0.28,4.29)	0.94 (0.21,4.19)	1.0	.063
CVS	a	1.32 (0.81,2.46)	0.67 (0.32,1.42)	0.56 (0.24,1.28)	0.97 (0.48,1.97)	1.0	.176
	b	1.68 (0.91,3.47)	0.84 (0.39,1.82)	0.74 (0.31,1.74)	1.07 (0.52,2.23)	1.0	.089
	c	1.77 (0.95,3.68)	0.91 (0.42,1.98)	0.77 (0.33,1.82)	1.13 (0.54,2.36)	1.0	.081
	d	2.05 (0.98,4.78)	0.86 (0.34,2.16)	0.88 (0.34,2.26)	1.13 (0.48,2.64)	1.0	.052

Males		<3.06	3.06‐	3.37‐	3.64‐	≥4.05	
Number		364	355	408	311	353	
Angina	a	1.23 (0.79,1.42)	0.81 (0.43,1.55)	0.43 (0.19,0.97)	0.38 (0.15,0.91)	1.0	.231
	b	1.30 (0.84,1.98)	0.88 (0.44,1.75)	0.41 (0.18,0.97)	0.67 (0.26,1.69)	1.0	.131
	c	1.31 (0.87,2.13)	0.93 (0.46,1.88)	0.44 (0.18,1.06)	0.70 (0.28,1.78)	1.0	.10
	d	1.49 (0.94,2.47)	1.14 (0.52,2.49)	0.50 (0.19,1.32)	0.37 (0.10,1.37)	1.0	.098
CAD	a	1.30 (0.85,2.19)	0.49 (0.20,1.16)	0.75 (0.36,1.56)	0.35 (0.13,0.95)	1.0	.087
	b	1.49 (0.92,3.10)	0.47 (0.19,1.18)	0.81 (0.37,1.76)	0.60 (0.20,1.72)	1.0	.093
	c	1.79 (0.94,3.83)	0.59 (0.23,1.50)	0.94 (0.42,2.07)	0.66 (0.22,1.92)	1.0	.082
	d	1.62 (0.95,2.81)	0.44 (0.15,1.29)	0.70 (0.28,1.72)	0.41 (0.11,1.51)	1.0	.110
Stroke	a	1.39 (0.93,3.05)	0.68 (0.19,2.40)	0.29 (0.06,1.43)	0.95 (0.28,3.13)	1.0	.102
	b	1.86 (0.95,5.51)	1.38 (0.42,4.46)	0.30 (0.06,1.55)	0.68 (0.18,2.52)	1.0	.096
	c	2.01 (0.96,7.09)	1.65 (0.49,5.56)	0.34 (0.10,1.75)	0.85 (0.22,3.27)	1.0	.061
	d	1.98 (0.98,5.95)	1.12 (0.27,4.55)	0.30 (0.10,1.63)	0.81 (0.19,3.41)	1.0	.076
CVS	a	1.26 (0.86,1.34)	0.46 (0.21,0.99)	0.77 (0.41,1.47)	0.36 (0.15,0.87)	1.0	.123
	b	1.90 (0.94,1.85)	0.45 (0.20,1.02)	0.84 (0.42,1.67)	0.63 (0.25,1.57)	1.0	.100
	c	1.95 (0.95,1.99)	0.48 (0.21,1.12)	0.89 (0.44,1.79)	0.66 (0.26,1.66)	1.0	.078
	d	1.96 (0.94,2.24)	0.54 (0.21,1.37)	1.08 (0.50,2.32)	0.34 (0.10,1.26)	1.0	.067

*Note:* a, crude; b, adjusted for age; c, adjusted for age and body mass; d, adjusted for age, body mass, physical activity (based on time spent on exercise and watching television), smoking status, alcohol intake, and socioeconomic status (2603 of 2711 of females and 1745 of 1791 of males).

**TABLE 3:** Odds ratios (95%CI) by birthweight quintiles for all participants (females and male), stratified by BMI

Birthweight, kg		<2.92	2.92‐	3.23‐	3.51‐	≥ 3.83	
Number		945	938	846	878	895	*P*
Angina	BMI <25	1.18 (0.72, 2.45)	0.75 (0.26, 2.19)	0.53 (0.13, 2.12)	0.77 (0.25, 2.36)	1.0	.451
	BMI 25‐29	1.59 (0.97, 3.27)	1.35 (0.68, 3.08)	0.74 (0.30, 1.84)	0.52 (0.21, 1.26)	1.0	.093
	BMI >30	1.76 (0.98, 3.07)	0.50 (0.15, 1.65)	0.66 (0.23, 1.85)	0.57 (0.21, 1.55)	1.0	.063
CAD	BMI <25	1.98 (0.65, 7.15)	1.53 (0.38, 6.11)	0.78 (0.13, 4.48)	0.22 (0.02, 2.02)	1.0	.324
	BMI 25‐29	1.94 (0.95, 2.16)	0.86 (0.38, 1.97)	0.49 (0.17, 1.42)	0.76 (0.31, 1.85)	1.0	.129
	BMI >30	1.97 (0.97, 3.95)	0.91 (0.25, 3.19)	0.94 (0.29, 3.03)	1.06 (0.36, 3.11)	1.0	.099
Stroke	BMI <25	1.31 (0.91, 3.7)	1.11 (0.53, 3.07)	1.62 (0.45, 3.51)	1.76 (0.34, 3.40)	1.0	.214
	BMI 25–29	1.37 (0.94, 3.10)	0.93 (0.33, 2.63)	0.90 (0.30, 2.59)	0.30 (0.06, 1.44)	1.0	.095
	BMI >30	1.72 (0.96, 4.66)	0.55 (0.10, 2.86)	0.95 (0.25, 3.53)	0.99 (0.27, 3.67)	1.0	.069
CVS	BMI <25	1.80 (0.92, 2.44)	0.74 (0.25, 2.16)	0.51 (0.13, 2.05)	0.77 (0.25, 2.34)	1.0	.109
	BMI 25‐29	1.91 (0.94, 3.08)	1.39 (0.66, 2.93)	0.71 (0.29, 1.74)	0.50 (0.21, 1.20)	1.0	.078
	BMI >30	1.95 (0.95, 3.10)	0.51 (0.15, 1.68)	0.79 (0.29, 2.12)	0.59 (0.22, 1.60)	1.0	.080

When we use the traditional definition of LBW (<2.5 kg), as shown in Table 4, the odds ratios (95%CI) for having angina, CAD, stroke and CVS, was higher among people with LBW, <2.5 kg, relative to those with normal birthweight, ≥2.5 kg. Females with LBW were at least 1.39, 1.40, 2.30, and 1.47 times more likely to have angina, CAD, stroke, and CVS compared to those ≥2.5 kg with *P* = .06, .19, .03, and .13, respectively. Similarly, males with LBW were 1.76, 1.48, 3.34, and 1.70 times more likely to have angina, CAD, stroke, and CVS compared to those ≥2.5 kg with *P* = .14, .13, .03, and .08, respectively.

**TABLE 4:** Odds ratios and 95%CI for angina, coronary artery disease (CAD), stroke, and all cardiovascular system abnormalities (CVS) among people with low birthweight

		Females	*P*	Males	*P*
Angina	a	1.39 (0.73, 2.62)	.147	1.76 (0.97, 3.95)	.069
	b	1.11 (0.58, 2.11)	.587	1.57 (0.77, 3.66)	.196
	c	1.10 (0.58, 2.11)	.599	1.62 (0.96, 3.78)	.099
	d	1.08 (0.54, 2.09)	.614	1.84 (0.91, 4.75)	.097
CAD	a	1.40 (0.79, 3.35)	.137	1.48 (0.77, 3.52)	.199
	b	1.11 (0.65, 2.71)	.621	1.29 (0.55, 3.13)	.424
	c	1.14 (0.66,2.81)	.577	1.37 (0.68, 3.37)	.327
	d	1.10 (0.32, 3.10)	.623	1.53 (0.74, 4.32)	.228
Stroke	a	2.30 (1.05, 5.07)	.037	3.34 (1.12, 10.01)	.031
	b	1.91 (0.96, 4.26)	.098	3.05 (0.99, 9.39)	.052
	c	1.91 (0.97, 4.23)	.099	3.31 (1.06, 10.3)	.039
	d	1.76 (0.93, 4.89)	.121	2.44 (0.97, 6.97)	.056
CVS	a	1.47 (0.90, 2.62)	.083	1.70 (0.86, 3.80)	.107
	b	1.32 (0.96, 2.12)	.109	1.50 (0.85, 3.48)	.134
	c	1.22 (0.85, 2.13)	.137	1.54 (0.86, 3.59)	.131
	d	1.09 (0.65, 2.11)	.435	1.76 (0.88, 4.52)	.104

*Note:* a, crude; b, adjusted for age; c, adjusted for age and body mass; d, adjusted for age, body mass, physical activity (based on time spent on exercise and watching television), smoking status, alcohol intake, and socioeconomic status (2603 of 2711 of females and 1745 of 1791 of males).

These relationships decrease in females and increased in males with adjustments for potential confounding factors.

The risk for having angina, CAD, stroke and CVS was decreased by 13%, 17%, 22% and 12% for each kilogram increase in birthweight. The odds ratios (95%CI) for having angina was 0.87 (0.75, 1.01), for CAD was 0.83 (0.75, 0.97), for stroke was 0.68 (0.48, 0.99), and for CVS was 0.88 (0.83, 0.96) for each kilogram increase in birthweight.

## 4 DISCUSSION

This study took advantage of the longitudinal population‐based resources of the AusDiab study to examine the associations of birthweight and angina, CAD, and stroke (CVA) in the general adult population. A significant association was identified between birthweight and angina, CHD, stroke and the overall CVS. People in the lowest birthweight quintile and those of LBW had higher risk than their higher birthweight people and the relationship was reinforced after adjustment for adult weight or body mass index and was independent of socioeconomic class. This applied in analyses of unadjusted data in females and significance relationship persisted with adjustment for the age and current body size for both females and males. Furthermore, the relationship persisted with adjustment for physical activity, smoking, alcohol intake, family history, and current socioeconomic status.

The Ausdiab‐birthweight is the first study of its kind to examine the effect of birthweight on the development of various chronic diseases in a representative adult population. We and others had shown that birthweight is inversely associated with CVD risk factors such as raised blood pressure, dyslipidaemia, diabetes, and metabolic syndrome/glucose intolerance.^35^, ^36^, ^37^, ^38^, ^39^, ^40^, ^41^, ^42^, ^43^, ^44^

A research was carried on people from Aberdeen, 1950s‐Prospective‐Children‐Cohort, during time when environmental conditions, as proved by low infant and mother death rates, were quite advantageous for both children and female. This research showed an inverse relationship between occurrence of CHD and CVA and their original weight at birth and the .^45^ The risk was like that described for Swedish female and male population that were born during 1915 to 1929. Among the 10 636 male during the study period, the hazard ratios for CHD fell with increasing birthweight size.^14^, ^15^ Many studies in both men and women in Europe, the United States, and India, reported similar association between LBW and CHD. These epidemiological, as well as ours, cohort studies have found a roughly 20% lower risk of CVD for every kilogram of higher birth weight.^46^

The inverse associations observed between birth weight and CHD is not markedly changed by adjustment for adult BMI.^46^ Although adjustment for BMI has little impact, stratification by BMI has indicated that birth weight and BMI may interact to predict risk of CHD. Weight gain increases the risk of CVD among all adults, but especially for those born small. Lube et al concluded that the acceleration of early infant weight gain may aggravate the effects of LBW. Multiple interactions between hemodynamic and metabolic parameters foreshadow the clustering of cardiometabolic risk factors later in life.^47^

The magnitude of the association was strong; it was independent of social class including educational level or income or type of inhabitant.^45^ The associations between birth weight and cardiovascular mortality have been shown to be independent of socioeconomic status at birth and during adulthood and of known adult lifestyle influences that might confound them (eg, cigarette smoking, diet, and exercise).^12^, ^13^, ^48^ Adult lifestyle, however, clearly adds to the effects of early life^12^, ^49^; for example, studies in Finland demonstrated that the highest incidence of CHD occurs among men who were thin at birth and also had low household incomes as adults.^49^ The results of randomized trials of nutritional interventions in infancy have led to the hypothesis that relative undernutrition and slower infant growth benefit later CVD.^50^, ^51^ Various CVD may be.

In our study, people who did not respond to the questionnaire, and those who could not recall their birthweight, were older and had higher rates of diabetes than those who reported a birthweight. Hence, overstatement of an exacerbating effect of lower birthweights on glycemic dysregulation in our study group is unlikely. Among birthweight respondents it is reassuring that the mean birthweight of those who guessed their birthweight was similar to those who obtained their birthweights from medical records or from a family member. This was also the case in British Telecom study.^52^ In addition, the mean recalled birthweight in our study, 3.37 (0.7) kg, is consistent with that reported in those born between 1931 and 1939 in Hertfordshire in the United Kingdom,^53^ with the Health Professional Follow Up Study.^54^

Our results are suggestive of the importance of LBW as a risk factor for CVDs that is well known in advance of any other risk factors that may develop later during life. Nelson et al found that arterial stiffening, and aging process, starts early in life and that arterial function and aging properties could be programmed during fetal life or influenced by adverse growth patterns in early postnatal life.^55^ Cardiovascular disorders may manifest early in life via various risk factors.^1^, ^2^, ^3^, ^5^, ^6^, ^56^ Lurbe et al found that children (mean age of 9.9 years) who had lower birth weights tended to have not only the highest blood pressure values but also the highest blood pressure variability, independent of the increases in ambulatory blood pressure values.^57^ Similarly, Lurbe et al disclosed a relationship between birth weight and ambulatory pulse pressure while seeking to advance knowledge about the possible associations between birth weight and cardiovascular risk.^58^ They also, reported that the results showed a relatively aged phenotype of large‐vessel function in the children with the lowest birth weights. These early alterations may be amplified throughout life and may contribute to the increased cardiovascular risk associated with LBW.^59^ Hence, Katsuragi et al reported that a LBW was associated with various cardiovascular risk factors including high low‐density lipoprotein and total cholesterol levels in men, and hypertension and diabetes mellitus in women aged 40 to 69.^60^ Low‐density lipoprotein (*P* < .05), and total cholesterol (*P* < .01) levels in men, and systolic (*P* < .05) and diastolic (*P* < .05) blood pressure in women were significantly inversely related to birth weight when controlling for age, body mass index, medication, and lifestyle.^60^ However, Lurbe et al assessed, in a prospective study, the association of birth weight (BW) and growth pattern on cardiometabolic risk factors in a cohort followed from birth to 10 years of age. They concluded that although BW was a proxy of the events during fetal life and projected its influence later, the influence of gaining weight was a key determinant in the risk to develop obesity and metabolic abnormalities.^61^

This phenomenon probably has more implications for the cardiovascular disorders in every country where LBWs is increasing and the newborns survive. The advancement in intensive care and medical care improved with time allowing lower birthweight infants to increasingly survive to adult life. In all populations, a worldwide secular trend toward higher levels of body fat and BMI potentially compounds the potentiation of other risk factors such as glycaemic abnormalities expression associated with lower birthweights. Modest increases in body fat might have a trivial impact on CVDs burden when acting in isolation, but substantial impact when other risk factors are also operating. It would be prudent, to adopt policies of intensified whole of life surveillance of lower birthweight people, anticipating this risk. Also, in more developed countries, LBW as the earliest known risk factor would add a value to the risk stratification for early identification of cardiac disease or its risk factors.^62^ This may guide the point of care decision for further testing and management selection that sets a platform for risk reduction based on biological platform stratification.^62^

### 4.1 Limitations

The study was conducted among Australian people and the study used a self‐recall questionnaire to obtain birthweight data. We opted for this method of obtaining birthweight as there are no readily available data banks of birthweights that cover the AusDiab study population. Many seminal studies, which have reported associations of birthweight with adult health, have employed this technique,^13^, ^52^, ^54^, ^63^ with response rates often less than described here. The British Telecom study had a 50% response rate and only 39.4% provided data on birthweight,^52^ the British Women's Heart and Health Study had a 60% response rate and 33% reported their birthweight^63^ and the Health Professional Follow‐up Study had a 75% response rate and 59% of the responders reported their birthweight.^54^

There are several additional limitations to the present study. First, there are two major sources of bias, first, the responder bias where 37% of participants did not provide their birthweight in this survey and second, the recollection bias where there is no verification that birthweight data, we accurate. Also, there was no correction for the gestational age. In addition, there are no data on maternal fetal complications that may have influenced health of participants beyond birthweight alone. Another limitation is the relatively small number for a cross‐sectional study and hence *P* value not always significant.

## CONFLICT OF INTEREST

Both authors declare no conflict of Interest related to the current manuscript.

## DISCLOSURE

The AusDiab study was approved by the Scientific Research Committee and certify that the study was performed in accordance with the ethical standards as laid down in the 1964 Declaration of Helsinki and its later amendments ethical standards.

## CONSENT FOR PUBLICATION

Both authors have agreed to the publication and to be accountable for all aspects of the work in ensuring that questions related to the accuracy or integrity of any part of the work are appropriately investigated and resolved.

## DATA AVAILABILITY STATEMENT

Data is part of the cross‐sectional cohort AusDiab study and not available publicly.

## References

[1] Al Salmi I , Hoy WE , Kondalsamy‐Chennakesavan S , et al. Disorders of glucose regulation in adults and birth weight: results from the Australian Diabetes, Obesity and Lifestyle (AUSDIAB) Study. Diabetes Care. 2008;31(1):159‐164. 17934149 10.2337/dc07-1170

[2] Al Salmi I , Shaheen M , Hannawi S . Birthweight and gestational age: early life management strategy to population health for non‐communicable diseases. Int J Pediatr Res. 2018;4(2):9.

[3] Al Salmi I , Hannawi S . Birth weight, gestational age, and blood pressure: early life management strategy and population health perspective. Saudi J Kidney Dis Transpl. 2019;30(2):299‐308. 31031365 10.4103/1319-2442.256836

[4] Al Salmi I , Hoy WE , Kondalsamy‐Chennakes S , Wang Z , Healy H , Shaw JE . Birth weight and stages of CKD: a case‐control study in an Australian population. Am J Kidney Dis. 2008;52(6):1070‐1078. 18640755 10.1053/j.ajkd.2008.04.028

[5] Al Salmi I , Hannawi S . Birth weight and gestational age: early life management strategy to population health for glucose disorders. Integr Obesity Diabetes. 2018;4(3):5.

[6] Al Salmi I , Hannawi S . Birth weight and gestational age: early life management strategy to population health for cardiac diseases. J Integr Cardiol. 2018;4(5):3.

[7] Barker DJ , Osmond C . Infant mortality, childhood nutrition, and ischaemic heart disease in England and Wales. Lancet. 1986;1(8489):1077‐1081. 2871345 10.1016/s0140-6736(86)91340-1

[8] Barker DJP . The origins of the developmental origins theory. J Intern Med. 2007;261(5):412‐417. 17444880 10.1111/j.1365-2796.2007.01809.x

[9] Syddall HE , Sayer AA , Simmonds SJ , et al. Birth weight, infant weight gain, and cause‐specific mortality: the Hertfordshire cohort study. Am J Epidemiol. 2005;161(11):1074‐1080. 15901628 10.1093/aje/kwi137

[10] Osmond C , Barker DJ , Winter PD , Fall CH , Simmonds SJ . Early growth and death from cardiovascular disease in women. BMJ. 1993;307(6918):1519‐1524. 8274920 10.1136/bmj.307.6918.1519 PMC1679586

[11] Eriksson JG , Forsen T , Tuomilehto J , Osmond C , Barker DJ . Early growth and coronary heart disease in later life: longitudinal study. BMJ. 2001;322(7292):949‐953. 11312225 10.1136/bmj.322.7292.949 PMC31033

[12] Frankel S , Elwood P , Sweetnam P , Yarnell J , Smith GD . Birthweight, body‐mass index in middle age, and incident coronary heart disease. Lancet. 1996;348(9040):1478‐1480. 8942776 10.1016/S0140-6736(96)03482-4

[13] Rich‐Edwards JW , Stampfer MJ , Manson JE , et al. Birth weight and risk of cardiovascular disease in a cohort of women followed up since 1976. BMJ. 1997;315(7105):396‐400. 9277603 10.1136/bmj.315.7105.396 PMC2127275

[14] Leon DA , Lithell HO , Vagero D , et al. Reduced fetal growth rate and increased risk of death from ischaemic heart disease: cohort study of 15 000 Swedish men and women born 1915‐29. BMJ. 1998;317(7153):241‐245. 9677213 10.1136/bmj.317.7153.241 PMC28614

[15] Hypponen E , Leon DA , Kenward MG , Lithell H . Prenatal growth and risk of occlusive and haemorrhagic stroke in Swedish men and women born 1915–29: historical cohort study. BMJ. 2001;323(7320):1033‐1034. 11691760 10.1136/bmj.323.7320.1033 PMC59382

[16] Lawlor DA , Davey Smith G , Ebrahim S . Birth weight is inversely associated with coronary heart disease in post‐menopausal women: findings from the British women's heart and health study. J Epidemiol Community Health. 2004;58(2):120‐125. 14729890 10.1136/jech.58.2.120 PMC1732671

[17] Eriksson JG , Forsen T , Tuomilehto J , Osmond C , Barker DJ . Early growth, adult income, and risk of stroke. Stroke. 2000;31(4):869‐874. 10753990 10.1161/01.str.31.4.869

[18] Barker DJ . Fetal origins of coronary heart disease. BMJ. 1995;311(6998):171‐174. 7613432 10.1136/bmj.311.6998.171 PMC2550226

[19] Barker DJ . In utero programming of cardiovascular disease. Theriogenology. 2000;53(2):555‐574. 10735050 10.1016/s0093-691x(99)00258-7

[20] Barker DJ , Osmond C , Forsen TJ , Kajantie E , Eriksson JG . Trajectories of growth among children who have coronary events as adults. N Engl J Med. 2005;353(17):1802‐1809. 16251536 10.1056/NEJMoa044160

[21] Bendtsen TF , Nyengaard JR . The number of glomeruli in type 1 (insulin‐dependent) and type 2 (non‐insulin‐dependent) diabetic patients. Diabetologia. 1992;35(9):844‐850. 1397779 10.1007/BF00399930

[22] Huxley R . Fatal flaw in the fetal argument. Br J Nutr. 2006;95(3):441‐442. 16512927 10.1079/bjn20051609

[23] Huxley R , Neil A , Collins R . Unravelling the fetal origins hypothesis: is there really an inverse association between birthweight and subsequent blood pressure? Lancet. 2002;360(9334):659‐665. 12241871 10.1016/S0140-6736(02)09834-3

[24] Huxley R , Owen CG , Whincup PH , Cook DG , Colman S , Collins R . Birth weight and subsequent cholesterol levels: exploration of the "fetal origins" hypothesis. JAMA. 2004;292(22):2755‐2764. 15585736 10.1001/jama.292.22.2755

[25] Lucas A , Fewtrell MS , Cole TJ . Fetal origins of adult disease‐the hypothesis revisited. BMJ. 1999;319(7204):245‐249. 10417093 10.1136/bmj.319.7204.245 PMC1116334

[26] Dunstan DW , Zimmet PZ , Welborn TA , et al. The Australian diabetes, obesity and lifestyle study (AusDiab)—methods and response rates. Diabetes Res Clin Pract. 2002;57(2):119‐129. 12062857 10.1016/s0168-8227(02)00025-6

[27] Cameron AJ , Welborn TA , Zimmet PZ , et al. Overweight and obesity in Australia: the 1999‐2000 Australian diabetes, obesity and lifestyle study (AusDiab). Med J Aust. 2003;178(9):427‐432. 12720507 10.5694/j.1326-5377.2004.tb05998.x

[28] Dunstan DW , Salmon J , Owen N , et al. Associations of TV viewing and physical activity with the metabolic syndrome in Australian adults. Diabetologia. 2005;48(11):2254‐2261. 16211373 10.1007/s00125-005-1963-4

[29] Dunstan DW , Zimmet PZ , Welborn TA , et al. The rising prevalence of diabetes and impaired glucose tolerance: the Australian diabetes. Obesity and Lifestyle Study Diabetes Care. 2002;25(5):829‐834. 11978676 10.2337/diacare.25.5.829

[30] Welfare AIoHa . The Active Australia Survey: A Guide and Manual for Implementation, Analysis and Reporting. Australia: Australia Institute of Health and Welfare; 2003:49.

[31] National Physical Activity Guidelines for Australians: Active Australia. In: Commonwealth Department of Health and Aged Care. Canberra A, Commonwealth Department of Health and Aged Care, editor. 1999.

[32] Salmon J , Owen N , Crawford D , Bauman A , Sallis JF . Physical activity and sedentary behavior: a population‐based study of barriers, enjoyment, and preference. Health Psychol. 2003;22(2):178‐188. 12683738 10.1037//0278-6133.22.2.178

[33] World Health Organization . Global nutrition targets 2025: low birth weight policy brief. World Health Organization, 2014 https://apps.who.int/iris/handle/10665/149020.

[34] World Health Organization . WHO Consultation on Obesity (‎1999: Geneva, Switzerland)‎ & World Health Organization. (‎2000)‎. Obesity: preventing and managing the global epidemic: report of a WHO consultation. World Health Organization Geneva: WHO; 1998 https://apps.who.int/iris/handle/10665/42330. 11234459

[35] Al Salmi I , Hoy W , Kondalsamy‐Chennakesavan S , Shaw J . Metabolic syndrome and birthweight‐ results from the AusDiasb survey. Diab Vasc Dis Res. 2005;2:161.

[36] Al Salmi I , Hoy W , Kondalsamy‐Chennakesavan S , Shaw J . Birth weight and blood pressure‐results from AusDiab survey. Nephrol Ther. 2005;10(3):A411.

[37] Al Salmi I , Hoy W , Wang Z , Gobe G , Barr E , Shaw J . Birthweight is inversely correlated with type 2 diabetes mellitus in the general population: results from the Ausdiab study. Diabet Med. 2006;23(s4):iv‐788.

[38] Al Salmi I , Hoy W , Wang Z , Gobe G , Barr E , Shaw J . Birthweight is inversely correlated with indices of glycemia in the general population: results from the Ausdiab study. Diabet Med. 2006;23(s4):iv‐788.

[39] Al Salmi I , Hoy W , Wang Z , Gobe G , Barr E , Shaw J . Lower birthweights pre‐dispose to the metabolic syndrome: results from the Ausdiab study. Diabet Med. 2006;23(s4):iv‐788.

[40] Al Salmi I , Hoy W , Wang Z , et al. Glucose disorders and birthweight‐results from the AusDiab study. Obes Rev. 2006;7(2):A0503.

[41] Huxley RR , Shiell AW , Law CM . The role of size at birth and postnatal catch‐up growth in determining systolic blood pressure: a systematic review of the literature. J Hypertens. 2000;18(7):815‐831. 10930178 10.1097/00004872-200018070-00002

[42] McKeigue PM , Lithell HO , Leon DA . Glucose tolerance and resistance to insulin‐stimulated glucose uptake in men aged 70 years in relation to size at birth. Diabetologia. 1998;41(10):1133‐1138. 9794098 10.1007/s001250051042

[43] Barker DJ , Eriksson JG , Forsen T , Osmond C . Fetal origins of adult disease: strength of effects and biological basis. Int J Epidemiol. 2002;31(6):1235‐1239. 12540728 10.1093/ije/31.6.1235

[44] Barker DJ , Martyn CN , Osmond C , Hales CN , Fall CH . Growth in utero and serum cholesterol concentrations in adult life. BMJ. 1993;307(6918):1524‐1527. 8274921 10.1136/bmj.307.6918.1524 PMC1679540

[45] Lawlor DA , Ronalds G , Clark H , Smith GD , Leon DA . Birth weight is inversely associated with incident coronary heart disease and stroke among individuals born in the 1950s: findings from the Aberdeen children of the 1950s prospective cohort study. Circulation. 2005;112(10):1414‐1418. 16129799 10.1161/CIRCULATIONAHA.104.528356

[46] Rich‐Edwards J . Epidemiology of the fetal origins of adult disease: cohort studies of birthweight and cardiovascular disease In: Langley‐Evans SC, ed. Frontiers in Nutritional Sciences: Fetal Nutrition and Adult Disease. Oxford: CAB International Press; 2004:87‐104.

[47] Lurbe E , Garcia‐Vicent C , Torro MI , Aguilar F , Redon J . Associations of birth weight and postnatal weight gain with cardiometabolic risk parameters at 5 years of age. Hypertension. 2014;63(6):1326‐1332. 24688125 10.1161/HYPERTENSIONAHA.114.03137

[48] Stein CE , Fall CH , Kumaran K , Osmond C , Cox V , Barker DJ . Fetal growth and coronary heart disease in South India. Lancet. 1996;348(9037):1269‐1273. 8909379 10.1016/s0140-6736(96)04547-3

[49] Barker DJ , Forsen T , Uutela A , Osmond C , Eriksson JG . Size at birth and resilience to effects of poor living conditions in adult life: longitudinal study. BMJ. 2001;323(7324):1273‐1276. 11731388 10.1136/bmj.323.7324.1273 PMC60299

[50] Singhal A , Cole TJ , Fewtrell M , Deanfield J , Lucas A . Is slower early growth beneficial for long‐term cardiovascular health? Circulation. 2004;109(9):1108‐1113. 14993136 10.1161/01.CIR.0000118500.23649.DF

[51] Singhal A , Lucas A . Early origins of cardiovascular disease: is there a unifying hypothesis? Lancet. 2004;363(9421):1642‐1645. 15145640 10.1016/S0140-6736(04)16210-7

[52] Davies AA , Smith GD , Ben‐Shlomo Y , Litchfield P . Low birth weight is associated with higher adult total cholesterol concentration in men: findings from an occupational cohort of 25,843 employees. Circulation. 2004;110(10):1258‐1262. 15326068 10.1161/01.CIR.0000140980.61294.4D

[53] Phillips DI , Goulden P , Syddall HE , et al. Fetal and infant growth and glucose tolerance in the Hertfordshire cohort study: a study of men and women born between 1931 and 1939. Diabetes. 2005;54(Suppl 2):S145‐S150. 16306332 10.2337/diabetes.54.suppl_2.s145

[54] Curhan GC , Willett WC , Rimm EB , Spiegelman D , Ascherio AL , Stampfer MJ . Birth weight and adult hypertension, diabetes mellitus, and obesity in US men. Circulation. 1996;94(12):3246‐3250. 8989136 10.1161/01.cir.94.12.3246

[55] Nilsson PM , Lurbe E , Laurent S . The early life origins of vascular ageing and cardiovascular risk: the EVA syndrome. J Hypertens. 2008;26(6):1049‐1057. 18475139 10.1097/HJH.0b013e3282f82c3e

[56] Al Salmi I , Hannawi S . Birthweight and Lipids in Adult Life: Population‐Based Cross Sectional Study. Lipids. 2020;55(4):365‐374. 10.1002/lipd.12242. 32372421

[57] Lurbe E , Torro I , Rodriguez C , Alvarez V , Redon J . Birth weight influences blood pressure values and variability in children and adolescents. Hypertension. 2001;38(3):389‐393. 11566910 10.1161/01.hyp.38.3.389

[58] Lurbe E , Torró I , Alvarez V , Aguilar F , Redon J . The impact of birth weight on pulse pressure during adolescence. Blood Press Monit. 2004;9(4):187‐192. 15311145 10.1097/00126097-200408000-00003

[59] Lurbe E , Torro MI , Carvajal E , Alvarez V , Redon J . Birth weight impacts on wave reflections in children and adolescents. Hypertension. 2003;41(3 Pt 2):646‐650. 12623973 10.1161/01.HYP.0000048341.52293.7C

[60] Katsuragi S , Okamura T , Kokubo Y , Ikeda T , Miyamoto Y . Birthweight and cardiovascular risk factors in a Japanese general population. J Obstet Gynaecol Res. 2017;43(6):1001‐1007. 28503828 10.1111/jog.13316

[61] Lurbe E , Aguilar F , Alvarez J , Redon P , Torro MI , Redon J . Determinants of Cardiometabolic risk factors in the first decade of life: a longitudinal study starting at birth. Hypertension. 2018;71(3):437‐443. 29358459 10.1161/HYPERTENSIONAHA.117.10529

[62] Maron BA , Maron JL , Abman SH . The case for bringing birthweight to adult cardiovascular medicine. Am J Cardiol. 2020;127:191‐192. 32402484 10.1016/j.amjcard.2020.04.015 PMC8211405

[63] Lawlor DA , Bedford C , Taylor M , Ebrahim S . Agreement between measured and self‐reported weight in older women. Results from the British women's heart and health study. Age Ageing. 2002;31(3):169‐174. 12006304 10.1093/ageing/31.3.169

